# Measuring impact of vaccination among wildlife: The case of bait vaccine campaigns for classical swine fever epidemic among wild boar in Japan

**DOI:** 10.1371/journal.pcbi.1010510

**Published:** 2022-10-06

**Authors:** Ryota Matsuyama, Takehisa Yamamoto, Yoko Hayama, Ryosuke Omori

**Affiliations:** 1 Veterinary Epidemiology Unit, Department of Preventive Veterinary Medicine, School of Veterinary Medicine, Rakuno Gakuen University, Ebetsu, Japan; 2 Division of Transboundary Animal Disease Research, National Institute of Animal Health, National Agriculture and Food Research Organization, Tsukuba, Japan; 3 Division of Bioinformatics, International Institute for Zoonosis Control, Hokkaido University, Sapporo, Japan; UNITED STATES

## Abstract

Understanding the impact of vaccination in a host population is essential to control infectious diseases. However, the impact of bait vaccination against wildlife diseases is difficult to evaluate. The vaccination history of host animals is generally not observable in wildlife, and it is difficult to distinguish immunity by vaccination from that caused by disease infection. For these reasons, the impact of bait vaccination against classical swine fever (CSF) in wild boar inhabiting Japan has not been evaluated accurately. In this study, we aimed to estimate the impact of the bait vaccination campaign by modelling the dynamics of CSF and the vaccination process among a Japanese wild boar population. The model was designed to estimate the impact of bait vaccination despite lack of data regarding the demography and movement of wild boar. Using our model, we solved the theoretical relationship between the impact of vaccination, the time-series change in the proportion of infected wild boar, and that of immunised wild boar. Using this derived relationship, the increase in antibody prevalence against CSF because of vaccine campaigns in 2019 was estimated to be 12.1 percentage points (95% confidence interval: 7.8–16.5). Referring to previous reports on the basic reproduction number (*R*_0_) of CSF in wild boar living outside Japan, the amount of vaccine distribution required for CSF elimination by reducing the effective reproduction number under unity was also estimated. An approximate 1.6 (when *R*_0_ = 1.5, target vaccination coverage is 33.3% of total population) to 2.9 (when *R*_0_ = 2.5, target vaccination coverage is 60.0% of total population) times larger amount of vaccine distribution would be required than the total amount of vaccine distribution in four vaccination campaigns in 2019.

## Author summary

Vaccination of wildlife is important to control infectious diseases in animals. However, the impact of common vaccination of wildlife, bait vaccination, is difficult to evaluate owing to difficulty in obtaining the vaccination history at the individual level. Mathematical modelling can estimate the impact of vaccination; however, the demography and movement of hosts are required to describe disease dynamics. In this study, we aimed to estimate the impact of bait vaccination by modelling the dynamics of classical swine fever (CSF) and the vaccination among Japanese wild boar. The model was designed to estimate the impact of bait vaccination despite lack of data regarding the demography and movement of wild boar. Using our model, the increase in antibody prevalence because of vaccination in 2019 was estimated to be 12 percentage points. Furthermore, we estimated the amount of vaccine distribution required for CSF elimination by reducing the effective reproduction number under unity. Referring to previous reports on the basic reproduction number of CSF in wild boar living outside Japan, it was estimated that an approximate 1.6 to 2.9 times larger amount of vaccine distribution would be required than the total amount of vaccine distribution in four vaccination campaigns in 2019.

## Introduction

Vaccination of wildlife is an important tool to control infectious diseases relevant to wild animals. In the procedures of vaccination against wildlife, bait vaccination is one of the widely accepted methods [1,2]. Bait vaccination is the oral immunisation using baits which can deliver the effective dose of vaccines. The decline of rabies incidences among wild carnivores [3] and that of classical swine fever (CSF) incidences among Eurasian wild boar (*Sus scrofa*) [4] by bait vaccination programmes are remarkable epidemiological examples that the bait vaccination has shown its effectiveness worldwide. The bait vaccination can be a practical tool for the control of wildlife diseases, and hence, raised the discussion on the optimal strategy of the bait vaccine distribution [1,2].

Measuring the impact of vaccination, i.e., vaccine effectiveness, is essential to develop the optimal strategy of bait vaccination. Generally, vaccination history at individual animal level is required to measure the vaccine effectiveness. For example, a common epidemiological measurement of vaccine effectiveness is (1 – odds ratio) × 100, which compares the odds of vaccination status among cases to that among controls [5]. However, in wildlife epidemiology, vaccination history at an individual level is often difficult to obtain under field conditions. Thus, the evaluation of vaccine effectiveness using vaccination history is difficult. In addition, wildlife hosts can acquire immunity against disease not only from vaccines but also a natural infection. To measure the impact of bait vaccination accurately, the antibody acquired by the bait vaccination should be distinguished from that acquired by a natural infection with the infectious disease. Thus, the establishment of method to estimate the impact of vaccination with a different approach from the traditional method is required in the lack of the data on history of immunity acquisition by vaccination/natural infection.

The use of a mathematical model describing the disease dynamics among hosts can be a potential solution to estimate the impact of vaccination in the lack of the data on vaccination history of hosts. The impact of vaccination against diseases has often been evaluated using the Susceptible–Infected–Recovery (SIR) model [6], which can be applied to the disease in wildlife populations. However, modelling the disease dynamics of wildlife diseases is often challenging due to the lack of data regarding host animals. For example, data on demography, movement, and disease dynamics in wildlife hosts are often unavailable. To address this issue, we developed a method to estimate the impact of bait vaccination without data regarding population size and movement of the hosts by utilising a time-series of the proportion of infected (reverse transcription-polymerase chain reaction [RT-PCR]-positive) and immunised (enzyme-linked immunosorbent assay [ELISA]-positive RT-PCR-negative) hosts. In the present study, we describe the method and demonstrate its application to example data of bait vaccination against CSF outbreak among Japanese wild boar.

As mentioned above, CSF among wild boar is an example that an infectious disease is controlled by bait vaccination against wildlife [7,8]. The distribution of live-attenuated bait vaccines has been recognised as an effective countermeasure to control CSF epidemics in wild boar. The scheme of bait vaccination using the CSFV C-strain vaccine was established during the 1990s in European countries [4,9]. The increase in the anti-CSF antibody by vaccination was confirmed by experimental infection in pigs and European wild boar [7,10,11]. In addition, the increase in the anti-CSF antibody associated with vaccination has been confirmed in field conditions. For example, Kaden et al. [9] reported an approximate 32 percentage-point increase (from 0.0% to 31.8%) in the proportion of immunised wild boar after a double-vaccination campaign (two vaccinations with a 2-week interval) in an area where CSF did not exist in Germany. In addition, Rossi et al. [12] compared the anti-CSF antibody prevalence in wild boar populations between vaccine-distributed and non-distributed areas in France. After three vaccination campaigns, they detected an approximate 40 percentage-point increase (approximately from 10% to 50%) in the antibody prevalence in wild boar populations. In Japan, since 2019, the distribution of the CSF bait vaccine (Pestiporc Oral, IDT Biologika GmbH, Dessau-Rosslau, Germany) has been implemented against re-emerged CSF in wild boar [13,14]. The proportion of immunised individuals changed from 4% to 74% (i.e., an approximate 70 percentage-point increase) among adult wild boar after a 1-year bait vaccine campaign [13].

In contrast to the reported increase in the immune wild boar, the impact of the bait vaccination campaign against CSF has not yet been well understood in the epidemiological context due to the difficulty of measuring vaccination history in wildlife. Although vaccination history can be traced using oxytetracycline [9] and iophenoxic acid [15] as biomarkers to differentiate between vaccinated and non-vaccinated individuals, the application of those biomarkers to wild boar as a food resource (game meat) is controversial in terms of food safety [16]. Using the Differentiating Infected from Vaccinated Animals (DIVA) vaccine for CSF may solve this problem [17], however, a DIVA type bait vaccine is not commercially available and is not commonly used as bait vaccines in field conditions.

In Japan, before 2018, no case of CSF in wild boar or domestic pigs had been reported for 26 years [18]. CSF re-emerged in 2018 in Gifu Prefecture, located in the central part of Honshu Island [13,18,19]. The first case of a CSF outbreak in domestic pigs was reported from a pig farm on 9 September 2018. Subsequently, a case of CSF in a wild boar was reported on 13 September 2018 by an active surveillance implemented by the Gifu Prefectural Government. The CSFV strain isolated from the index case of the domestic pig showed clade-level genetic differences with other strains from past CSF outbreaks in Japan [20]. The 26 years absence of CSF cases and the results of genetic analysis strongly suggested the re-introduction of CSFV from outside of Japan. As the epidemic area expanded gradually, the Gifu Prefectural Government began its first bait vaccine campaign on 26 March 2019. Four bait vaccine campaigns were implemented from March 2019 to October 2019; a total of 24,001 units, 28,110 units, 35,920 units, and 34,880 units of bait vaccine were distributed between 26 March and 29 March, 11 April and 11 May, 10 July and 16 July, and 20 August and 24 August, respectively. The area where the bait vaccine was distributed expanded due to the progression of CSF epidemics.

In the present study, we aimed to estimate the impact of bait vaccination among Japanese wild boar by developing a mathematical model that describes the disease dynamics of CSF in a wild boar population under the lack of data regarding host animals. In the case of CSF in Japanese wild boar, data on population size, population dynamics, and movement of hosts are not available. We developed a method to estimate the impact of bait vaccination without data regarding population size and movement of the hosts by utilising a time-series of the proportion of infected and immunised wild boar. Using our model, we also aimed to quantify the amount of vaccines required to control the CSF epidemic.

## Results

**Fig 1:**
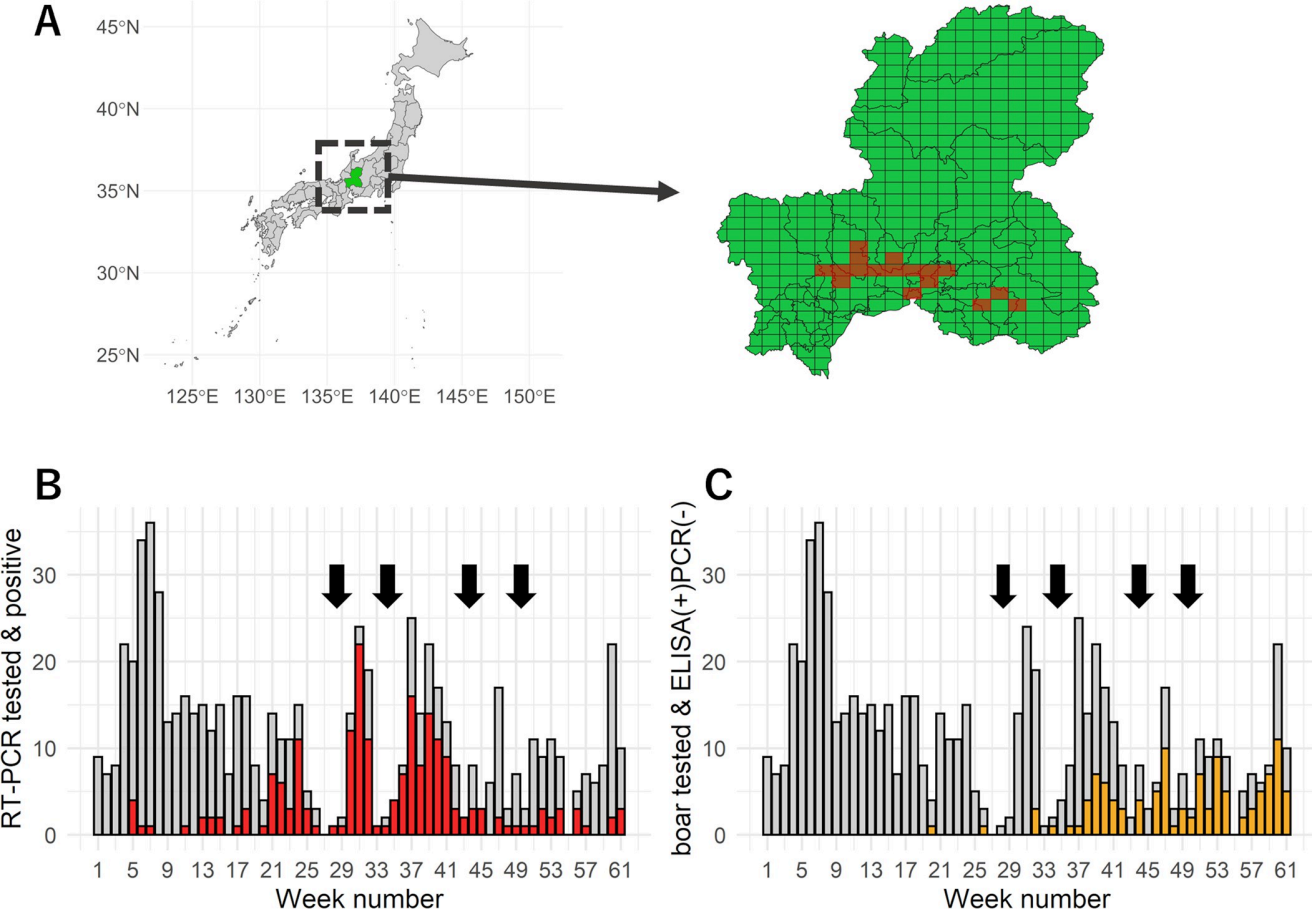
**(A) The location of Gifu Prefecture in Japan and selected grids.** The location of Gifu Prefecture in Japan (green) and the selected grids (17 grids; red) are shown. Map base layer of Japan was obtained from Natural Earth (http://www.naturalearthdata.com/about/terms-of-use/) and is available at https://www.naturalearthdata.com/http//www.naturalearthdata.com/download/10m/cultural/ne_10m_admin_0_countries_jpn.zip. Map base layer of Gifu Prefecture was obtained from the digital national land information of Japan (https://nlftp.mlit.go.jp/index.html) and is available at https://nlftp.mlit.go.jp/ksj/gml/datalist/KsjTmplt-N03-v3_0.html#prefecture21. **(B) Time-series change in the number of boar tested that resulted in PCR-positive from week 1 to week 61**. The red and grey bars demonstrate the number of PCR(+) and PCR(-) boar reported between week 1 and week 61, respectively. Arrows denote the vaccine campaigns. **(C) Time-series change in the number of boar tested that resulted in ELISA-positive and PCR-negative from week 1 to week 61.** The orange bar demonstrates the number of ELISA(+)PCR(-) boar reported between the week 1 and week 61. The grey bar demonstrates the sum of the number of ELISA(+)PCR(+) boar and that of ELISA(-) boar reported between the week 1 and week 61. Arrows denote the vaccine campaigns. ELISA, enzyme-linked immunosorbent assay; PCR, polymerase chain reaction; RT, reverse transcription.

We constructed a Susceptible–Exposed–Infected–Recovered (SEIR) model that describes the transmission dynamics of CSF and the population dynamics of wild boar (see Materials and Methods section). Using the model, we analysed the epidemiological data of CSF outbreak in Gifu Prefecture, Japan. The data were collected by the Gifu Prefectural Government through active surveillance, which started soon after the detection of first case of CSF in domestic pigs in September 2018. The data of bait vaccine distribution in Gifu Prefecture were also used for our analyses. Setting the week from 13^th^ September 2018 to 19^th^ September 2018 as the first week, we rearranged the data into weekly time-series data. To avoid the bias from the heterogeneity in frequencies of investigation and vaccine distribution, we extracted the area where wild boar were investigated and were vaccinated routinely with the same frequency over our research period (see the detail of criteria written in Materials and Methods section). Setting a 4.6 km × 5.6 km rectangular grid as a geographical grid for our analyses, 17 grids were selected and defined as our research area (Fig 1A).

Wild boar specimens sampled in the surveillance were tested for ELISA and RT-PCR. The result of ELISA demonstrated the presence/absence of acquired immunity against CSFV in tested wild boar while the result of PCR illustrates the presence/absence of the CSFV gene in tested wild boar. Based on the test results, we defined the wild boar with i) test result positive for ELISA and test result negative for PCR (hereinafter, ELISA(+)PCR(-)) as immune individuals by recovery or vaccination and ii) test result positive for PCR regardless of the result of the ELISA test (hereinafter, PCR(+)) as infected individuals.

The weekly numbers of wild boar that were tested for ELISA and PCR, immunised individuals, and infected individuals are shown in Fig 1B and 1C. In total, 694 wild boar were tested for ELISA and PCR. The weekly number of immunised wild boar ranged from 0 (weeks 1–19, 21–25, 27–31, 33, 35, 43, 45, 55) to 11 (week 60), and a total of 114 wild boar were recorded. The weekly number of infected wild boar ranged from 0 (weeks 1–4, 8–10, 12, 16, 19, 27, 46, 55, 58–59) to 22 (week 31), resulting in a total of 201 wild boar. The number of vaccines distributed in the selected grids during the four vaccination campaigns were as follows: 8,633 units the first time (weeks 28–29), 8,130 units the second time (weeks 34–35), 2,960 units the third time (week 44), and 2,590 units the fourth time (weeks 49–50).

**Fig 2:**
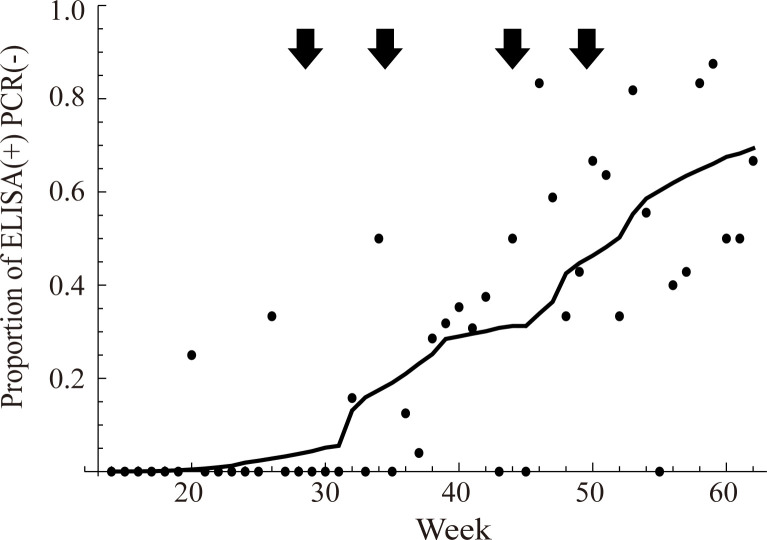
Model fitting with the observed proportion of ELISA(+)PCR(-) in the tested boar. We fit the model describing (*R*_*i*_(*t*)+*V*_i_ (*t*))/*N*_i_(*t*) by maximising the likelihood shown in Eq 18. The mean estimated proportion of ELISA(+) PCR(-) individual is demonstrated by a solid line. Each dot denotes the observed value of the proportion of ELISA(+)PCR(-) individuals in each week. Arrows denote the vaccine campaigns.

Using the time-series data, we estimated the recovery rate from CSF infection, *γ*, the lethality rate by CSF, *μ*_d_. Using the estimates of *γ* and *μ*_d_, case fatality ratio (hereinafter, CFR) of CSF among the wild boar was estimated. CFR is generally defined as the proportion of deaths from a certain disease (in this study, CSF) among total infected cases (infected wild boar, PCR(+)), and theoretically defined as CFR = lethality rate/(lethality rate + recovery rate + natural mortality rate) according to the method described by Omori et al. [21] and Matsuyama et al. [22]. We assumed that the natural mortality rate (*μ*) is constant and following to a reported value, 0.15 per year [23]. The maximum likelihood estimates of the recovery rate from CSF infection, *γ*, and the lethality rate by CSF infection, *μ*_d_, were estimated to be 0.002 (95% confidence intervals [CI]: 0.000–0.006) per week and 0.149 (95% CI: 0.128–0.168) per week, respectively. The CFR of CSF was calculated from the estimates of the recovery rate and the lethality rate, resulting in a value of 0.964 (95% CI: 0.899–0.985). The delay in the vaccination effect, *τ*, was estimated to be 4 weeks (95% CI: 4–5), and the vaccine efficacy per vaccine unit, *k*, was estimated to be 0.065 (95% CI: 0.041–0.088). We confirmed that the observed time-series of the sum of the proportion of the immunised boar and the recovered boar (ELISA(+)PCR(-)) is well explained by the estimated parameters (Fig 2). *R*^2^ (squared Pearson’s correlation coefficient between observed data and model prediction) was calculated as a measurement of the goodness-of-fit for observed versus estimated weekly proportions of immunised boar (*R*^2^ = 0.57). In addition, a leave-one-out cross-validation was conducted, and *R*^2^ was calculated for the observed versus estimated proportions of immunised boar (*R*^2^ = 0.56). Furthermore, we validated our model by comparing it with a model hypothesising no vaccination impact (see Materials & Methods). Akaike information criterion (AIC [24]) of our model and the model hypothesising no vaccination impact was 88.6 and 91.1, respectively, and the goodness-of-fits of these models were significantly different (likelihood ratio test, *p* = 0.04).

**Fig 3:**
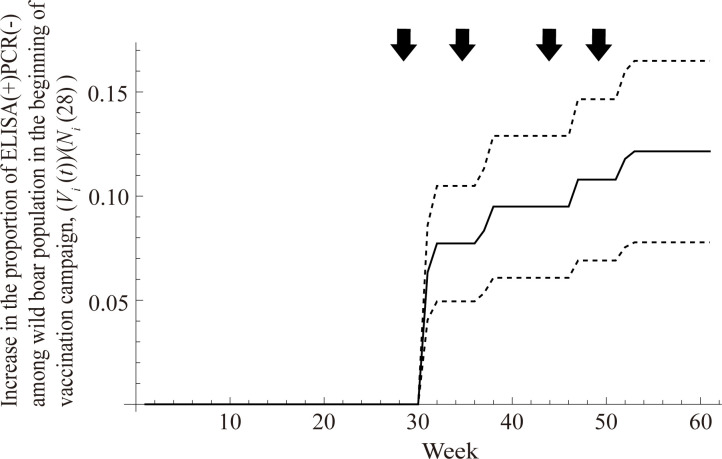
Change in the proportion of immunised wild boar by the bait vaccination among the total wild boar population at the beginning of vaccination campaign, (*V*_i_ (*t*))/(*N*_i_(28)). Mean estimated increment of the proportion of ELISA(+) PCR(-) is demonstrated by the solid line. The 95% confidence intervals are shown by dashed lines. The bait vaccination was implemented in the weeks 28–29, weeks 34–35, week 44, and weeks 49–50. Arrows denote the timing of vaccine campaigns.

The increase in the proportion of immunised wild boar by bait vaccination was also estimated (Fig 3). The mean proportion of ELISA(+)PCR(-) increased by 7.7 percentage points after the first distribution (weeks 32–33, from 0.0% to 7.7%), 1.8 percentage points after the second distribution (weeks 34–39, from 7.7% to 9.5%), 1.3 percentage points after the third distribution (week 48, from 9.5% to 10.8%), and by 1.4 percentage points after the fourth distribution (weeks 53–54, from 10.8% to 12.1%). As a result, the total percentage-point increment of the ELISA(+)PCR(-) proportion was estimated to be 12.1 (95% CI: 7.8–16.5).

**Fig 4:**
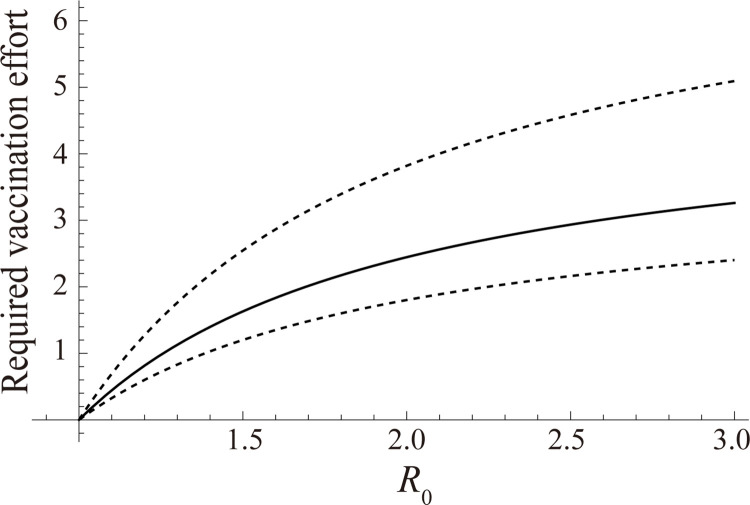
Required vaccination effort for CSF elimination at week 28 compared to the cumulative vaccination effort until week 50 [*V*_eff,i_(50)] with a varied basic reproduction number. Mean estimated vaccination effort for the elimination of CSF is demonstrated by the solid line. The dashed lines denote 95% confidence intervals of the estimated values.

The required effort of bait vaccination to achieve elimination of CSF was estimated in comparison with the impact of vaccination if all vaccines distributed during the four vaccine campaigns were distributed at the first vaccine campaign in week 28 (Fig 4). If all vaccines distributed in the week 28, the expected percentage point increase in the proportion of ELISA(+)PCR(-) was estimated to be 20.4% (95% CI: 13.1–27.6). The required effort compared to the scenario if all vaccines distributed in the week 28 were estimated to be 1.6 (95% CI: 1.2–2.5) times, 2.4 (95% CI: 1.8–3.8) times, and 2.9 (95% CI: 2.2–4.6) times, when the *R*_0_ is 1.5, 2.0, and 2.5, respectively. Results of sensitivity analyses with respect to variations in the natural mortality rate, population growth rate, and condition of data extraction are shown in S1–S3 Figs.

## Discussion

We developed a framework to estimate the impact of vaccination among wildlife despite a lack of data regarding the demography and movement of host. To this end, we developed a mathematical model describing the transmission dynamics of infectious diseases and identifying the proportion of immune hosts by recovery process and vaccination to appropriately estimate the impact of vaccination. As a case study, we applied our framework with the outbreak of CSF among wild boar in Japan. Our method demonstrated that the impact of bait vaccination can be estimated from the time-series data of captured and tested hosts, infected hosts, and immune hosts, even if the data regarding population size and movement of the host are lacking. Bait vaccination has been implemented not only in CSF, but also in many diseases involving wildlife hosts (such as rabies in wild canids [20] and tuberculosis in wild animals [25,26]). Our method is applicable to any wildlife/zoonotic disease if migration is negligible.

To our knowledge, this is the first study to estimate the impact of bait vaccination on CSF in a Japanese wild boar population. The increase of the proportion of immunised wild boar by the cumulative vaccination effort of four vaccine campaigns was estimated as 12.1 percentage points (from 0.0% to 12.1%), and the required effort to eliminate CSF epidemics compared to the cumulative impact of four vaccination campaigns were estimated to be approximately 1.6 to 2.9 times greater.

The estimates of epidemiological parameters in the present study agree with those of previous studies. The estimate of the lethality rate from CSF, 0.149 per week, falls within the range of previously reported values for European wild boar, 0.021 per week to 0.562 per week [7]. Additionally, the estimate was included in the 95% CI of the estimated lethality rate in our previous study (0.165 per week with 0.081–0.250 as 95% CI), which was estimated for the same Japanese wild boar population before vaccination by using a different analytical method [22]. The estimates of recovery rate and CFR in the present study, 0.002 per week and 0.964, respectively, were also close to the estimates from our previous study (recovery rate, 0.004 per week; CFR, 0.959) [22]. Our estimates of a recovery rate were lower than reported values in European wild boar, which ranged from 0.01 per week [27] to 0.416 per week (calculated from a daily recovery rate, 1/[13 days]; an inverse of the infectious period [13 days] described in Table 1 in the “Annex B. Models” section of European Food Safety Authority [7]), suggesting low recovery and low survival rates of infected wild boar in Japan. This can be explained by the difference in the virulence of the CSFV strain detected from European wild boar and that from Japanese wild boar [22]. The CSFV in Japan is reported as a moderately-virulent strain [28], suggesting that the virus can cause a longer infectious period compared with the high virulent strains reported in European countries [22,29]. Theoretically, as the inverse of the infectious period is equivalent to the recovery rate, the longer infectious period of CSFV in Japan can cause a lower recovery rate among Japanese wild boar.

**Table 1:** Descriptions of parameters for the estimation.

Symbol	Description	Value	Reference
*γ*	Recovery rate from CSF per week	Estimated	Estimated
*μ*_*d*_	CSF-induced lethality rate per week	Estimated	Estimated
*μ*	Natural mortality rate per year	0.15	Natural mortality rate of European wild boar was estimated by Toïgo et al. [23]
*r*_*y*_	population growth rate per year	1.6	Mean value of yearly population growth rates among Japanese wild boar. We calculated the mean value from the estimates between 2004 and 2011 reported by Matsumoto et al. [44]
*k*	Efficacy of bait vaccine per one unit of vaccine	Estimated	Estimated
*τ*	Time delay *τ* from the vaccine distribution to immunisation	Estimated	Estimated

To date, several strategies to control CSF among wild boar other than bait vaccination have been considered. For instance, movement restriction and culling of wild boar have been attempted in European countries [8]. However, implementation of movement restriction and culling is often difficult, and their effectiveness is considered limited [4,8]. The main habitat of wild boar is forest. Forests cover more than 66% of the land of Japan, resulting more than twice as high as the forest coverage of some European countries [13,30]. Also, the forests in Japan are highly continuous compared with those in European countries which tends to be fragmented and independent [13,31]. Furthermore, most of the forests in Japan located in and around steep mountainous area [13,32]. This landscape condition makes the movement restriction and culling difficult. Therefore, bait vaccination is considered the most practical control strategy in Japan [13], although its effectiveness has not been evaluated quantitatively. Providing quantitative evidence for the effectiveness of bait vaccination among Japanese wild boar is an imperative task.

The present study provides quantitative evidence that bait vaccination increased the antibody prevalence against CSF among Japanese wild boar. The percentage point increase of immunised wild boar if all vaccines were distributed at the first vaccination campaign was approximately 20% (increased from 0% to 20.4%). This value is approximately 1/3 and 3/5 of the target proportion of immunised individuals when *R*_0_ is assumed to be 2.5 and 1.5, respectively (Fig 4). The immunised proportion required for CSF elimination is reachable if: 1) vaccine effectiveness is not saturated even if a large amount of bait vaccine is distributed; and 2) there is enough financial/working capacity for additional vaccine distribution. The detailed dose responses of bait vaccines should be clarified for appropriate vaccine campaigns [12]. In addition, the financial/working costs of vaccine distribution and the maintenance of herd immunity in wild boar by vaccination must be discussed carefully.

In the previous studies, the impact of vaccination has been measured in CSF-free areas [9,12,33]. Kaden et al. [9] reported a 39.8 percentage-point increase, from 0.0% to 39.8%, after four vaccine campaigns (two double-vaccination campaigns) using the C-strain vaccine in Germany during 1993 Autumn to 1994 Spring. Rossi et al. [12] reported an approximately 40 percentage-point increase, from approximately 10% to 50%, after six vaccine campaigns (three double-vaccination campaigns) using the C-strain vaccine in France during 2004 Summer to 2005 Summer. Feliziani et al. [33] reported a 33.3 percentage-point increase, from 0.0% to 33.3%, after two vaccine campaigns (a double-vaccination campaign) using CP7-E2alf strain vaccine in Italy in 2012. The difference in the impact of vaccination may reflect the different conditions in terms of the situation of CSF epidemic in the area where vaccine was distributed (CSF-free or not), number of vaccination campaigns, vaccination strategy, vaccine strain, the strain of CSFV, landscape structure of the research area, population size of wild boar, and possible biological difference between European and Japanese wild boar [4,34–37].

To interpret our results regarding the required vaccination effort for CSF elimination, it should be noted that the estimated efforts can be applicable if the herd immunity threshold is accomplished in a short-term vaccine campaign. It may not be applicable for a long-term vaccination campaign (a few years) due to the maternally derived antibody and the immunity waning. Regarding the maternally derived antibody, it is detected in European wild boar piglets until they reach 2–3 months old [37,38]. Since the piglets of European wild boar aged less than 4.5 months old are poorly attracted by vaccine baits [7,12,34], it can be considered that the maternally derived antibody does not interfere with the antibody acquisition by bait vaccination in the field condition in European countries. However, as for Japanese wild boar, neither the duration of the maternally derived antibody nor the vaccine-intake behaviour of piglets has been understood. If the piglets of Japanese wild boar are more likely to incept the vaccine baits or have longer duration of maternally derived antibody compared with European wild boar, it may interfere with the effect of vaccination. Regarding the waning of immunity, vaccine-induced immunity by the CSFV C-strain in boar has been considered one that ‘might be even life-long’ [4]. However, the period of observation was limited (approximately 10 months [11]), and there is room for the impact of waning immunity on the effectiveness of long-term vaccine campaigns. The impact of maternally derived antibodies in piglets and waning immunity should be taken into account for the analysis of long-term vaccination campaigns.

In addition to the limitations described above, this study has several limitations. First, we assumed an equality of sampling rate in any state of *S*, *E*, *I*, *R*, and *V* in the wild boar population. If an infected wild boar is less likely to be captured due to the lethality of CSF, the assumption is violated. The sampling process for captured wild boar should be studied for future clarification. Second, the data on found-dead wild boar were not included in our analysis due to the nature of the surveillance of found-dead boar. The application of found-dead data is difficult due to the potential sampling bias. For example, the heterogeneity in frequencies of carcass lost (e.g., decomposition and removal by scavengers) differs the detection rate of dead body [39,40]. The loss of carcass depends on season and environment, which can result the difference in the detection rate of dead body over time and between areas. Also, the public awareness, interest, and human population density can change the reporting rate of found-dead [39]. In data used in this study, the found-dead surveillance was conducted through the reporting of dead wild boar by local community. Thus, the reporting rate of found-dead wild boar can be different over time and between areas due to change in the public awareness, interest, and human population density. These biases are unique to the sampling of found-dead, and hence, (live-) captured boar and found-dead boar cannot be merged simply. Indeed, we observed the large temporal variance in the sampling of found-dead wild boar (S4 Fig), indicating the difficulty for the merge of the two data. If the biases in detection and reporting process of dead wild boar are quantified, and the random sampling of dead wild boar can be conducted, it can help to obtain more accurate estimate. Third, we could not include age-dependent heterogeneity of wild boar in our model because we did not have reliable data on the age of the captured wild boar. CSF severity in piglets is higher than in adults in wild boar, as observed in domestic pigs [4,41]. In addition, an age-specific difference in vaccine-intake behaviour has been reported in wild boar [12,42]. The age structure of wild boar populations should be analysed and incorporated to better estimate the epidemiological parameters and vaccination effects in the future. Fourth, the results of the present study were obtained from a limited area and research period. During the research period, most CSF outbreaks were concentrated in this area. When data are accumulated, the research area will be expanded. Fifth, we assumed that the natural mortality is constant during CSF outbreak. However, this assumption may be violated if the decrease in wild boar population by the CSF outbreak in contrast to the constant environmental resource results the decrease in natural mortality of wild boar (i.e., compensation between the CSF-induced mortality and natural mortality, as suggested by Artois [41]). The relationship between the natural mortality and the CSF-induced mortality among Japanese wild boar should be studied and clarified.

*R*^2^ was employed as a measurement of the goodness-of-fit for the observed data versus the model prediction. In addition, a cross-validation was conducted, and *R*^2^ was calculated for the observed data versus the model prediction. These *R*^2^ values indicate that our model captured the general trend in the observed data. The results of sensitivity analyses with respect to variations in the natural mortality rate, population growth rate, and the condition of data extraction suggests that our estimation was robust (S1–S3 Figs). Furthermore, we validated our model by comparing it with a model hypothesising no vaccination impact. The lower AIC of our model than that of the model hypothesising no vaccination impact demonstrates that our model has better predictability. Additionally, a likelihood ratio test showed that this difference in predictabilities was significant.

The present study demonstrated a simple method for estimating the impact of bait vaccination in wildlife in the absence of data regarding population size, dynamics, and movement of the hosts. Using our method, the increase in immunised wild boar by bait vaccination was detected quantitatively. Even if our method is applied, the implementation of continuous surveillance to collect time-series data of disease dynamics is important to quantify the impact of vaccination. Although continuous surveillance of a wildlife disease takes cost and effort, the collection of time-series data regarding the disease is worthwhile to control wildlife/zoonotic diseases.

## Materials and methods

### Mathematical model for the CSF transmission dynamics

In our previous study, we constructed a SEIR-based model by considering the population dynamics of wild boar [22]. Using the model and the dataset of proportions of infected and recovered individuals among tested wild boar, we estimated the lethality rate and recovery rate from CSF during the non-vaccinated period [22]. In the present study, we expanded our previous model to take the impact of bait vaccination into account. In the model, we considered the CSF disease dynamics, population dynamics of wild boar, and impact of bait vaccination in a certain geographical grid (hereinafter, grid), *i*, and at a certain time point, *t*. In this study, we set the spatial scale of grid *i* defined as approximately 4.6 km × 5.6 km and the unit of time *t* as 1 week, respectively (for the detail, see *Data selection* section). The disease dynamics of CSF and the population dynamics of wild boar can be modelled with the following equations:

$$\frac{dS_{i}}{dt}=\mu_{0}(t)N_{i}(t)-S_{i}(t)\sum_{j}\left(\beta_{ji}I_{j}(t)\right)-\mu S_{i}(t)-v(t)S_{i}(t),$$ (1)

$$\frac{dE_{i}}{dt}=S_{i}(t)\sum_{j}\left(\beta_{i,j}I_{j}(t)\right)-(\mu +\varepsilon )E_{i}(t)-v(t)E_{i}(t),$$ (2)

$$\frac{dI_{i}}{dt}=\varepsilon E_{i}(t)-(\mu +\gamma +\mu_{d})I_{i}(t),$$ (3)

$$\frac{dR_{i}}{dt}=\gamma I_{i}(t)-\mu R_{i}(t),$$ (4)

$$\frac{dD_{i}}{dt}={\mu N_{i}(t)+\mu }_{d}I_{i}(t),$$ (5)

$$\frac{dV_{i}}{dt}=v(t)(N_{i}(t)-I_{i}(t)-R_{i}(t)-V_{i}(t))-\mu V_{i}(t),$$ (6)

$$N_{i}(t)=S_{i}(t)+E_{i}(t)+I_{i}(t)+R_{i}(t)+V_{i}(t),$$ (7)

$$\frac{dN_{i}}{dt}=(\mu_{0}(t)-\mu )N_{i}(t)-\mu_{d}I_{i}(t).$$ (8)

where *S*_*i*_(*t*), *E*_*i*_(*t*), *I*_*i*_(*t*), *R*_*i*_(*t*), *D*_*i*_(*t*), and *V*_*i*_(*t*) represent the number of susceptible, latent, infectious, recovered, dead, and vaccinated individuals in grid *i* at calendar time *t*, respectively. *N*_*i*_(*t*) represents the total number of living wild boar in grid *i* at time *t*, that is, the population size of wild boar in grid *i* at time *t*. The parameters *β*_*ji*,_ *ε*, *γ*, *μ*_0_(*t*), *μ*, *μ*_*d*_, and *v*(*t*) denote the transmission rate of CSF from grid *j* to grid *i*, the transition rate from latent to infectives, recovery rate from the disease, birth rate of wild boar at time *t*, natural mortality rate, CSF-induced lethality rate, and immunisation rate by bait vaccination at time *t*, respectively. Note that when *j* ≠ *i*, parameter *β*_*ji*_ denotes the transmission rate by temporal movement of the wild boar from grid *j* to *i*. Since the wild boar is a sedentary species [43], long-term movement (migration) is rare. We assumed that migration was negligible in our analysis. As for *v*(*t*), we assumed that the distributed bait vaccines proportionally immunised wild boar so that the bait vaccine is efficacious even with a single oral intake [4,7]. Considering the time delay *τ* from the vaccine distribution to immunisation, we modelled the immunisation rate *v*(*t*) as *v*(*t*) = *kv*_*d*_(*t-τ*), where *k* is the parameter that denotes the change in the proportion of immunised wild boar by one unit of bait vaccine (i.e., efficacy of bait vaccine per one unit of vaccine) and *v*_*d*_(*t*) is the number of distributed bait vaccine at time *t*. We assumed that the time delay of the effect of vaccination, *τ*, includes not only the time from the vaccine distribution, oral intake, to the acquisition of immunity (usually 1–2 weeks after ingestion of baits [11]), but also the average time to capture and test a vaccinated animal.

Immune boar can be considered to acquire immunity from natural CSFV infection (recovered, *R*_*i*_(*t*) in our model) or from bait vaccination (vaccinated, *V*_*i*_(*t*) in our model), therefore the number of immune boar at time *t* in grid *i* was *R*_*i*_(*t*)+ *V*_*i*_(*t*). We assumed vaccinated state *V* was immunised by only vaccination through the intake of bait vaccines. We also assumed no maternally derived antibody in piglets in this study. The dynamics of the immune wild boar at time *t* can be written as:

$${\left(\frac{R_{i}(t)+V_{i}(t)}{N_{i}(t)}\right)}^{\prime }=\frac{{\left(R_{i}(t)+V_{i}(t)\right)}^{\prime }N_{i}(t)-(R_{i}(t)+V_{i}(t))N_{i}^{\prime }(t)}{{N_{i}(t)}^{2}}.$$ (9)

From Eqs (4), (6), and (8), Eq (9) is written as:

$${\left(\frac{R_{i}(t)+V_{i}(t)}{N_{i}(t)}\right)}^{\prime }=\frac{\left(\gamma I_{i}(t)-\mu R_{i}(t)+v(t)(N_{i}(t)-I_{i}(t)-R_{i}(t)-V_{i}(t))-\mu V_{i}(t))N_{i}(t)-(R_{i}(t)+V_{i}(t))((\mu_{0}(t)-\mu )N_{i}(t)-\mu_{d}I_{i}(t)\right)}{{N_{i}(t)}^{2}},$$ (10)

and can be simplified as:

$${\left(\frac{R_{i}(t)+V_{i}(t)}{N_{i}(t)}\right)}^{\prime }=v(t)\left(1-(\frac{I_{i}(t)}{N_{i}(t)}+\frac{R_{i}(t)+V_{i}(t)}{N_{i}(t)})\right)+\gamma \frac{I_{i}(t)}{N_{i}(t)}+\mu_{d}\frac{I_{i}(t)}{N_{i}(t)}\frac{R_{i}(t)+V_{i}(t)}{N_{i}(t)}-\mu_{0}(t)\frac{R_{i}(t)+V_{i}(t)}{N_{i}(t)}.$$ (11)

Solving Eq (11), we have:

$$\frac{R_{i}(t)+V_{i}(t)}{N_{i}(t)}=\frac{R_{i}(0)+V_{i}(0)}{N_{i}(0)}+\int_{0}^{t}\left[v(s)(1-(\frac{I_{i}(s)}{N_{i}(s)}+\frac{R_{i}(s)+V_{i}(s)}{N_{i}(s)}))+\gamma \frac{I_{i}(s)}{N_{i}(s)}+\mu_{d}\frac{I_{i}(s)}{N_{i}(s)}\frac{R_{i}(s)+V_{i}(s)}{N_{i}(s)}-\mu_{0}(t)\frac{R_{i}(s)+V_{i}(s)}{N_{i}(s)}\right]ds.$$ (12)

Note that the right side of Eq (12) consists of the proportion of immune boar [(*R*_*i*_(*t*)+*V*_*i*_(*t*))/*N*_*i*_(*t*)], that of infected wild boar [*I*_*i*_(*t*)/*N*_*i*_(*t*)], rate of immunisation by bait vaccination at time *t* [*v*(*t*)], recovery rate (*γ*), CSF-induced lethality rate (*μ*_*d*_), and birth rate [*μ*_0_(*t*)]. Even if: 1) the exact wild boar population size, and 2) wild boar transmission by movement between grids are not available, the right side of Eq (12) can be calculated by the proportions of infected and immune boar. Epidemiological parameters [recovery rate (*γ*) and lethality rate (*μ*_*d*_)], vaccine efficacy [*k* in *v*(*t*)], and the time delay of the vaccine effect (*τ*) can be estimated if data on the proportion of immune boar, infected individuals, and birth rates are available.

### Parameterisation of birth rate

We parameterised the birth rate from population growth rate per year of Japanese wild boar, *r*_y_, as reported by Matsumoto et al. [44]. When there is no CSF outbreak, the population dynamics of the wild boar can be written as:

$$\frac{dN_{i}}{dt}=(\mu_{0}(t)-\mu )N_{i}(t).$$ (13)

Solving Eq (15), we have:

$$\frac{N_{i}(t)}{N_{i}(0)}=exp(\int_{s=0}^{t}\mu_{0}(s)ds-\mu t).$$ (14)

Setting 1 week as a unit time, and assuming 1 year has 52 weeks, we have *r*_*y*_ = *N*_*i*_(52)/*N*_*i*_(0). Since the main birth season of Japanese wild boar was reported as May to June [45], we assumed that the birth season of the targeted wild boar population was between 3 May 2019 and 3 July 2019 (8 weeks, from week 34 to week 41 in our dataset) and a constant birth rate during the period ($\bar{\mu_{0}}$), i.e.,

$$\mu_{0}(t)=\left\{ \begin{array}{c} 0\;t<34,41<t\\ \bar{\mu_{0}}\;34\leq t\leq 41 \end{array}\right.,$$ (15)

and therefore,

$$r_{y}=exp(8\bar{\mu_{0}}-\mu ).$$ (16)

To obtain $\bar{\mu_{0}}$, we parameterised *r*_*y*_ as the average of *r*_*y*_ between 2002 and 2011 = 1.6 [19], and the natural mortality rate per year *μ* = 0.15 (approximately 0.003 per week) [23]. Solving Eq (16), we can obtain the birth rate as $\bar{\mu_{0}}$ = 0.079 per week.

### Estimation of parameters and case fatality ratio

As described later, the dataset used in the present study was reported in a discrete time interval (week). Therefore, we discretise Eq (12) as:

$$\frac{R_{i}(t)+V_{i}(t)}{N_{i}(t)}=\frac{R_{i}(0)+V_{i}(0)}{N_{i}(0)}+\sum_{s=0}^{t}\left[v(s)(1-(\frac{I_{i}(s)}{N_{i}(s)}+\frac{R_{i}(s)+V_{i}(s)}{N_{i}(s)}))+\gamma \frac{I_{i}(s)}{N_{i}(s)}+\mu_{d}\frac{I_{i}(s)}{N_{i}(s)}\frac{R_{i}(s)+V_{i}(s)}{N_{i}(s)}-\mu_{0}(t)\frac{R_{i}(s)+V_{i}(s)}{N_{i}(s)}\right].$$ (17)

The parameters used for estimation and their values are listed in Table 1. We assume that the sampling process of the immune wild boar follows a binomial process. The likelihood function describing the sampling process of immune wild boar can be written as:

$$\prod_{t}pmf(Bin(N_{data,i,t},\left(R_{i}(t)+V_{i}(t)\right)/N_{i}(t)),R_{data,i,t}+V_{data,i,t}),$$ (18)

where pmf(Bin (*n*, *p*), *x*) represents the probability mass function of the binomial distribution with the trial number *n* and probability *p* conditioned on observation *x*, *N*_*data*,*i*,*t*_ denotes the time-series data on the total number of captured and tested wild boar in grid *i* at time *t*, *R*_*data*,*i*,*t*_ denotes that of recovered wild boar in grid *i* at time *t*, and *V*_*data*,*i*,*t*_ denotes that of vaccinated wild boar in grid *i* at time *t*. For the estimation of *μ*_*d*_, *γ*, *k*, and *τ*, the likelihood function is maximised. Profile likelihood-based 95% CIs were calculated as 95% CI of the estimates.

From the estimates of *μ*_*d*_ and *γ*, CFR of CSF was determined by CFR = *μ*_*d*_/(*γ*+*μ*+*μ*_*d*_) [21]. The 95% CI of the CFR was calculated using parametric bootstrap resampling.

### CSF surveillance and bait vaccination of wild boar in Gifu Prefecture, Japan

Surveillance of CSF epidemics in wild boar and vaccinations was conducted by the Gifu Prefectural Government. Blood specimens were collected from captured wild boar. Capturing of wild boar was carried out by trapping and shooting by permitted hunters. Blood specimens of wild boar were tested by the public veterinary health service of Gifu Prefecture. RT-PCR tests for the CSFV gene and an ELISA test for the antibody against CSFV were carried out according to the protocol provided by the Ministry of Agriculture, Forestry and Fisheries (MAFF) [46]. The date and location (longitude and latitude) of captured wild boar were also recorded.

The site where the bait vaccine was distributed (hereinafter, vaccination site) was determined by the Gifu Prefectural Government. The Gifu Prefectural Government followed the guideline provided by MAFF; the guideline for distribution of the oral bait vaccine against classical swine fever [47], which recommends the vaccination to the sites i) close to the pig farm for the reduction in the risk of CSFV infection in the pig farm, ii) in and around the habitat of wild boar (e.g., resting site and/or feeding site), iii) where easily be accessed and monitored by the practitioner of vaccination, and iv) where easily be accepted to stakeholders (e.g., farmers working/residents living near the vaccination site). Once a place was set to be a vaccination site, feed for wild boar around the site (e.g., cones, rice brans) was distributed for baiting wild boar before vaccine distribution. The number of vaccination sites were 600, 937, 1796, and 1810 in the first, the second, the third, and the fourth vaccine campaigns, respectively at the whole prefecture-level. The number of distributed vaccine baits per site were 32–45 units, 30 units, 20 units, and 10–20 units in the first, the second, the third, and the fourth vaccine campaigns, respectively.

The distributed baits were collected 5 days after distribution to check for the loss of vaccines. The dates of distribution and collection, and geographical coordinates of the distribution points were recorded by Gifu Prefectural Government.

### Data selection

We obtained data on disease surveillance and bait vaccine campaigns from the Gifu Prefectural Government. The data were transformed for application to our model. We set a unit of the hunting grid designated by the Gifu Prefecture as a spatial grid for our analysis. The hunting grid divides the area between 136° 7’ 30” E and 137° 52’ 30” in longitude and between 35° 0’ 0” N and 36° 34’ 60” N in latitude into 28 × 36 grids (approximately 4.6 km × 5.6 km per grid). The weekly number of tested wild boar, the PCR-positive wild boar regardless of the results of the ELISA test (infected boar), and PCR-negative and ELISA-positive wild boar (recovered and vaccinated boar) were aggregated in each grid by setting the first week (week 1) from 13 September 2018 (the day when the initial case of CSF in wild boar was found) to 19 September 2018. For our data analysis, we used the data from week 1 to week 61 (13 September 2018 to 13 November 2019) to remove the impact of a substantial decline in sampling activity after week 61.

As CSF spread in wild boar, the area subjected to surveillance and vaccination was expanded. As a result, the frequencies of investigation and vaccine distribution varied among the hunting grids. To avoid the bias from the heterogeneity in frequencies of investigation and vaccine distribution, we extracted the grids where wild boar were investigated and were vaccinated routinely at the same frequency over the time period included in this study. To this end, we set two conditions for the extraction of grids: 1) the grids where wild boar were continuously captured and tested for RT-PCR and ELISA every 12 weeks between week 1 and week 60; and 2) the grids where all four vaccination campaigns occurred by 24 August 2019. All data were processed using R version 4.0.3 [48].

### Estimating the impact of bait vaccination and required effort of vaccine distribution for CSF control

We calculated the proportion of ELISA(+)PCR(-) among wild boar populations at the beginning of vaccination campaigns (i.e., wild boar population size in the 28^th^ week, *N*(28)) to evaluate the impact of the vaccination campaign. Using the data of distributed vaccines *v*_d_ and the estimates of *k* and *τ* (see the section “Estimation of parameters and case fatality ratio”), we calculated the change in the proportion of ELISA(+)PCR(-) among wild boar population at the beginning of vaccination campaign, *V*_*i*_(*t*)/*N*_*i*_(28), as follows:

$$\frac{V_{i}(t)}{N_{i}(28)}=\sum_{s=28}^{t}\left[\frac{N_{i}(s)}{N_{i}(28)}kv_{d}(t-\tau )(1-(\frac{I_{i}(s)}{N_{i}(s)}+\frac{R_{i}(s)+V_{i}(s)}{N_{i}(s)}))\right].$$ (19)

Relative population size *N*_*i*_(*s*)/*N*_*i*_(28) was obtained by numerically-solving Eq (8) with the estimate of *μ*_d_. The 95% CI of *V*_*i*_(*t*)/*N*_*i*_(28) was estimated using the 95% CI of *k*.

To estimate the required vaccination effort, we considered the herd immunity threshold of CSF. The herd immunity threshold has been defined using the basic reproduction number *R*_0_ (the average number of secondary cases that will be generated from an infectious individual in a completely susceptible population) [49]. With a known *R*_0_, the effective reproduction number under vaccination, *R*_v_, can be calculated as *R*_v_ = *R*_0_(1-*v*_c_), where *v*_c_ is the proportion of immunised wild boar by vaccination. An epidemic will become extinct if *R*_v_ satisfies below unity. From this relation, the herd immunity threshold to satisfy *R*_v_ < 1 is generally equated as *v*_c_ = 1-1/*R*_0_. Using the known number of distributed vaccines, the required vaccination effort at time *t* in grid *i*, *V*_eff,*i*_(*t*), is calculated as *V*_eff,*i*_(*t*) = *v*_c_/*V*_*i*_(*t*)/*N*_*i*_(*t*).

Referring to reported values of *R*_0_ in CSF among Eurasian wild boar outside Japan, which ranged from 1.1 to 2.8 [27,50,51], we assumed that the plausible value of *R*_0_ in CSF in Japanese wild boar can range between 1.0 and 3.0. Focusing on the cumulative impact of four vaccine campaigns that were completed in week 50, we estimated the required vaccination effort at week 28 compared to the cumulative vaccination effort until week 50 [*V*_eff,*i*_(50)] by varying the value of *R*_0_. All computations for the parameter estimation and the required vaccination effort were performed using Mathematica ver. 12.1.1.0 [52].

### Goodness-of-fit

To assess how well our model fits the data, we calculated *R*^2^, which is squared Pearson’s correlation coefficient between observed data and the model prediction. We also calculated the *R*^2^ value between the observed and estimated weekly proportions of immunised wild boar from week 1 to week 61. We also calculated *R*^2^ using the leave-one-out cross-validation. The leave-one-out cross-validation was conducted as follows: i) the data on the proportion of immunised wild boar at one random time point were removed from its time-series; ii) parameters were estimated using the remaining data; iii) the proportion of immunised wild boar at the time point corresponding to the removed data was predicted, and the model predictions were pooled; iv) the steps from i) to iii) were iterated 1,000 times, and v) an *R*^2^ value was calculated for the pooled observed versus predicted values.

### Model comparison

We performed a model comparison to assess the predictability of our model. We compared our model with a model hypothesising that the vaccination has no effect on immunisation (i.e., *k* = 0). A likelihood ratio test was conducted to test the difference in goodness-of-fit between the models. We also calculated the AIC for each model and compared the values.

### Sensitivity analysis

Our model includes several hypotheses. To assess the robustness of our estimation regarding required vaccination efforts, sensitivity analyses with respect to the natural mortality rate, population growth rate, and condition of data extraction were conducted. We varied the natural mortality rate and population growth rate from 0.07 to 0.23 and from 1.2 to 2.0, respectively. In terms of the sensitivity analysis with respect to the condition for data extraction, we explored different criteria in terms of test frequency, i.e., grids where boar were tested at least every 9–13 weeks.

## Supporting information

S1 Fig Sensitivity analysis with respect to *μ*. The required vaccination effort was estimated by varying the yearly natural mortality rate *μ* from 0.07 to 0.23 (baseline value = 0.15). Mean estimated vaccination effort for the elimination of CSF is demonstrated by the solid lines. The dashed lines denote 95% confidence intervals of the estimated values.
(EPS)

S2 Fig Sensitivity analysis with respect to *r*_*y*_. The required vaccination effort was estimated by varying the yearly growth rate of wild boar *r*_*y*_ from 1.2 to 2.0 (baseline value = 1.6). Mean estimated vaccination effort for the elimination of CSF is demonstrated by the solid lines. The dashed lines denote 95% confidence intervals of the estimated values.
(EPS)

S3 Fig Sensitivity analysis with respect to the condition for data extraction. The required vaccination effort was estimated by varying the data-extraction criteria in terms of the test frequency against CSF. The grids where at least one test was enrolled by every 9, 10, 11, 12, and 13 weeks were analysed (baseline value = 12). Mean estimated vaccination effort for the elimination of CSF is demonstrated by the solid lines. The dashed lines denote 95% confidence intervals of the estimated values.
(EPS)

S4 Fig Time-series change in the number of boar captured and found dead from week 1 to week 61. The black bar demonstrates the number of dead boar found between week 1 and week 61. The grey bar demonstrates the number of boar captured between the week 1 and week 61.
(TIFF)
