# Supplementary figures and images for: Measuring impact of vaccination among wildlife: The case of bait vaccine campaigns for classical swine fever epidemic among wild boar in Japan
Source: PLoS Comput Biol. 2022 Oct 6;18(10):e1010510. doi: 10.1371/journal.pcbi.1010510 (PMC9536577; doi:10.1371/journal.pcbi.1010510)

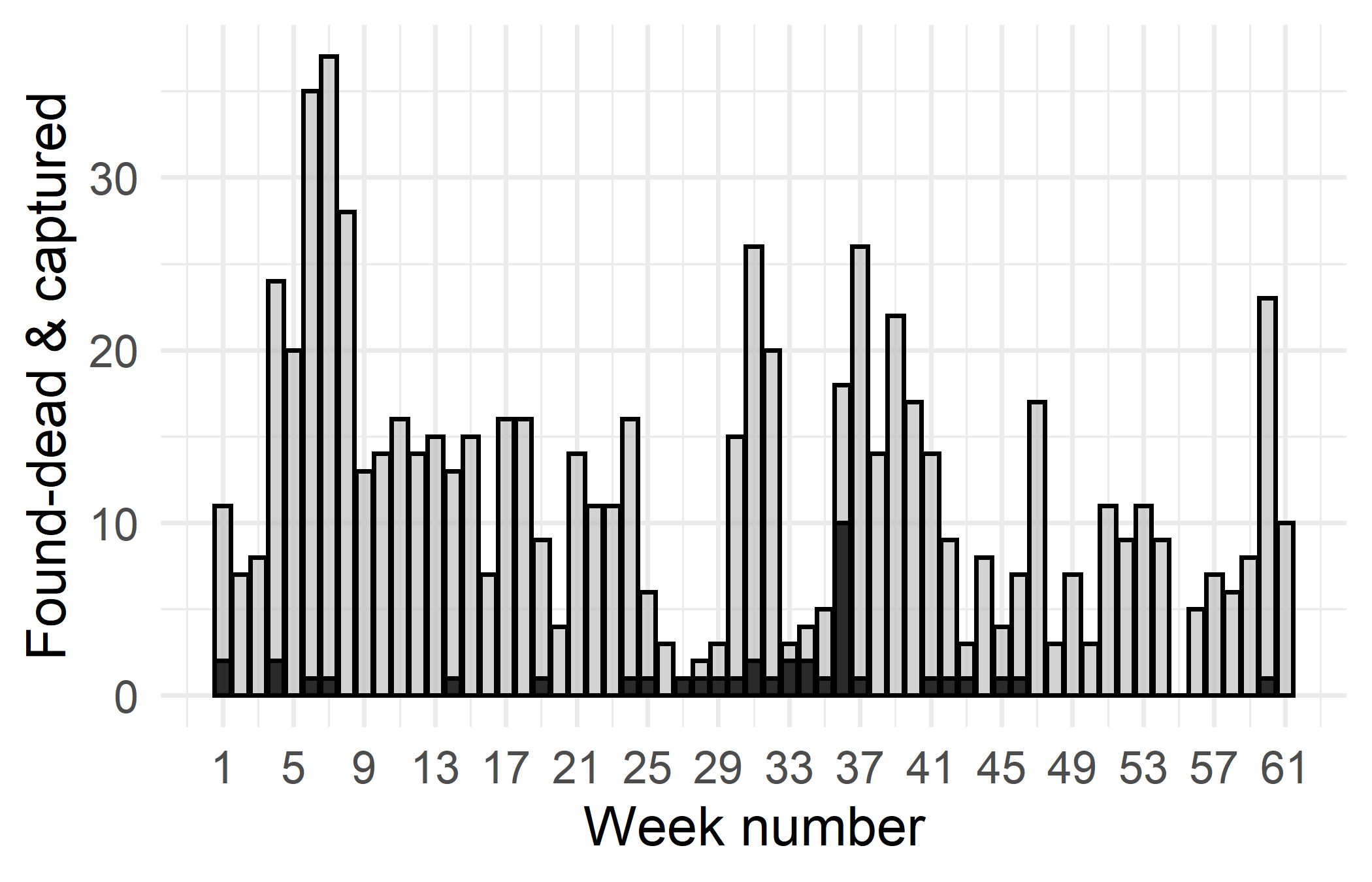

Supplement: S4 Fig — The black bar demonstrates the number of dead boar found between week 1 and week 61. The grey bar demonstrates the number of boar captured between the week 1 and week 61. (TIFF) [file pcbi.1010510.s004.tiff]
